# The three-dimensional structure of aspergilloglutamic peptidase from *Aspergillus niger*

**Published:** 2004-09-01

**Authors:** Hiroshi Sasaki, Atsushi Nakagawa, Tomonari Muramatsu, Megumi Suganuma, Yoriko Sawano, Masaki Kojima, Keiko Kubota, Kenji Takahashi, Masaru Tanokura

**Affiliations:** *1) Department of Biophysics and Biochemistry, Graduate School of Science, The University of Tokyo, 7-3-1 Hongo, Bunkyoku, Tokyo 113-0033, Japan; *2) Tokiwa Junior College, 1-430-1 Miwa, Mito, Ibaraki 310-8585, Japan; *3) Institute for Protein Research, Osaka University, 3-2 Yamadaoka, Suita, Osaka 565-0871, Japan; *4) Biophysics Division, National Cancer Center Research Institute, 5-1-1 Tsukiji, Chuo-ku, Tokyo 104-0045, Japan; *5) Department of Applied Biological Chemistry, Graduate School of Agricultural and Life Sciences, The University of Tokyo, 1-1-1 Yayoi, Bunkyo-ku, Tokyo 113-8657, Japan; *6) School of Life Science, Tokyo University of Pharmacy and Life Science, 1432-1 Horinouchi, Hachioji, Tokyo 192-0392, Japan

**Keywords:** Aspergilloglutamic peptidase, catalytic residue, glutamic peptidase, three-dimensional structure, X-ray crystallography

## Abstract

Aspergilloglutamic peptidase from *Aspergillus niger* is a novel pepstatin-insensitive acid endopeptidase distinct from the well-studied aspartic peptidases, and thus is an interesting target for protein structure/function studies. In the present study, we have determined the three-dimensional structure of the enzyme by X-ray crystallography to a 1.4-Å resolution. The results revealed that the enzyme has a unique structure, composed of two seven-stranded anti-parallel ***β***-sheets which form a ***β***-sandwich structure and appear to have a partial two-fold symmetry, suggesting its possible evolution by gene duplication and that the glutamic acid-110 and glutamine-24 in the heavy chain form a catalytic dyad, consistent with our results obtained by site-directed mutagenesis.

## Introduction

Aspergilloglutamic peptidase (AGP; *MEROPS* ID: G01.002; formerly called aspergillopepsin II) is an acid endopeptidase produced by the fungus *Aspergillus niger* var. *macrosporus*,1),2) belonging to the newly established family of glutamic peptidases (i.e., peptidase family G1). AGP is unique among several homologous peptidases in that it is the only two-chain structure enzyme among them.3) Moreover, it is completely different in amino acid sequence from the well-studied aspartic peptidases and is insensitive to aspartic peptidase-specific inhibitors, such as pepstatin, diazoacetyl-D,L-norleucine methyl ester/Cu^2+^ ions and 1,2-epoxy-3-(*p*-nitrophenoxy)propane. Furthermore, our recent site-directed mutagenesis studies4)–6) have demonstrated that two specific residues, the glutamic acid-110 (Glu B110) and the glutamine residue-24 (Gln B24), in the heavy chain are indispensable for the catalytic activity, different from the cases of aspartic peptidases which have a catalytic dyad of two aspartic acid residues.

To establish that these two residues (Glu B110 and Gln B24) are actually the catalytic residues, it is essential to elucidate the three-dimensional structure of the enzyme. Thus, we have solved the structure of AGP by X-ray crystallography to a 1.4-Å resolution. The results revealed that the enzyme has a unique structure, with an apparent partial two-fold symmetry, where the essential glutamic acid (Glu B110) and glutamine (Gln B24) residues identified by site-directed mutagenesis apparently form a catalytic dyad. Meanwhile, Fujinaga *et al*.7) have reported the three-dimensional structure of scytalidoglutamic peptidase (SGP or eqolisin; *MEROPS* ID: G01.001; formerly called scytalidopepsin B) at 2.1-Å resolution, which is almost identical with the structure of AGP. This has accelerated publication of our results in a preliminary form.

## Materials and methods

AGP was purified from the crude enzyme mixture obtained from the culture medium of *Aspergillus niger* var. *macrosporus*.2) The crystals were grown from AGP (100 mg/ml) solution containing 1.4 M ammonium sulfate, 5%(v/v) dimethyl sulfoxide, 50 mM glycine buffer (pH 2.1) by hanging-drop vapor diffusion method essentially as described.8),9) The crystals belonged to space group *P*2_1_2_1_2_1_ with unit cell dimensions of *a* = 55.1 Å, *b* = 70.7 Å and *c* = 38.3 Å, and had one molecule of AGP per asymmetric unit. All diffraction data were collected at BL6A in Photon Factory of High Energy Accelerator Research Organization (Tsukuba, Japan). The wavelength of X-rays used was 1.00 Å and the temperature was 10 °C. Data were processed using the HKL package10) and the CCP4 suite11) for the native protein data set, and WEIS12) for the derivative data sets. The crystal structure was solved by a multiple isomorphous replacement with anomalous scattering (MIRAS) method using K_2_PtCl_4_ and HgCl_2_ derivatives. The phases were calculated at 2.0-Å resolution with program MLPHARE11) in the CCP4 program suite resulting in the figure of merit 0.57. Subsequently, density modification was applied by solvent flattering and histogram matching using program DM.11) The initial model was constructed by using program O13) and refined with program X-PLOR.14) The current model contains the residues A1-32 (light chain) and B3-173 (heavy chain), 128 water molecules and 5 SO_4_^2−^ ions. The crystallographic *R*-factor is 19.7% (free *R*-factor is 22.6%) for 29,266 reflections in the 10.0-1.4-Å resolution range. The root-mean square deviations from ideality were 0.008 Å for bond lengths and 1.669 degree for bond angles. All figures were produced by the program PyMOL.15) Further experimental details will be published elsewhere, and the coordinates of AGP will soon be deposited in the Protein Data Bank.

## Results and discussion

**Fig. 1:**
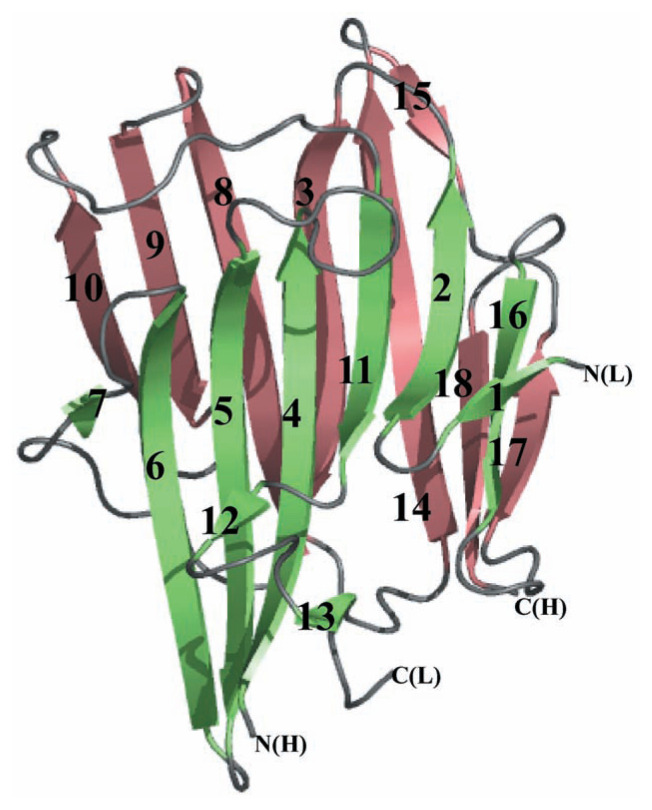
Overall structure of AGP. The structure is shown in a ribbon model. The ***β***-strands are numbered from the N-terminus to the C-terminus, starting from the light chain followed by the heavy chain. The first seven-stranded ***β***-sheet and two extra short strands 7 and 13 are shown in green and the second seven-stranded ***β***-sheet is in red. N(L) and C(L) indicate the N- and C-terminus, respectively, of the light chain, and N(H) and C(H) those of the heavy chain.

Figure 1 shows the overall structure of AGP in a ribbon model. The enzyme molecule was shown to be composed of eighteen ***β***-strands and the loops connecting them, being entirely devoid of ***α***-helix. This is consistent with the secondary structure prediction made previously from the circular dichroism spectroscopy.16) There are two seven-stranded anti-parallel ***β***-sheets (shown in green and red). These two sheets overlap with each other, thus forming a ***β***-sandwich structure. This unique structure is essentially almost the same as that of SGP7) and has not been reported previously in any other proteins. The C-terminus of the light chain is located close to the N-terminus of the heavy chain, consistent with the removal of the eleven residue intervening peptide during conversion of the proenzyme to the mature enzyme. The intervening peptide presumably protrudes from the rest of the protein molecule in the proenzyme so as to be easily susceptible to proteolysis at the two boundaries between the light chain C-terminus and the intervening peptide N-terminus and between the intervening peptide C-terminus and the heavy chain N-terminus. The function of this intervening peptide remains to be elucidated.

**Fig. 2:**
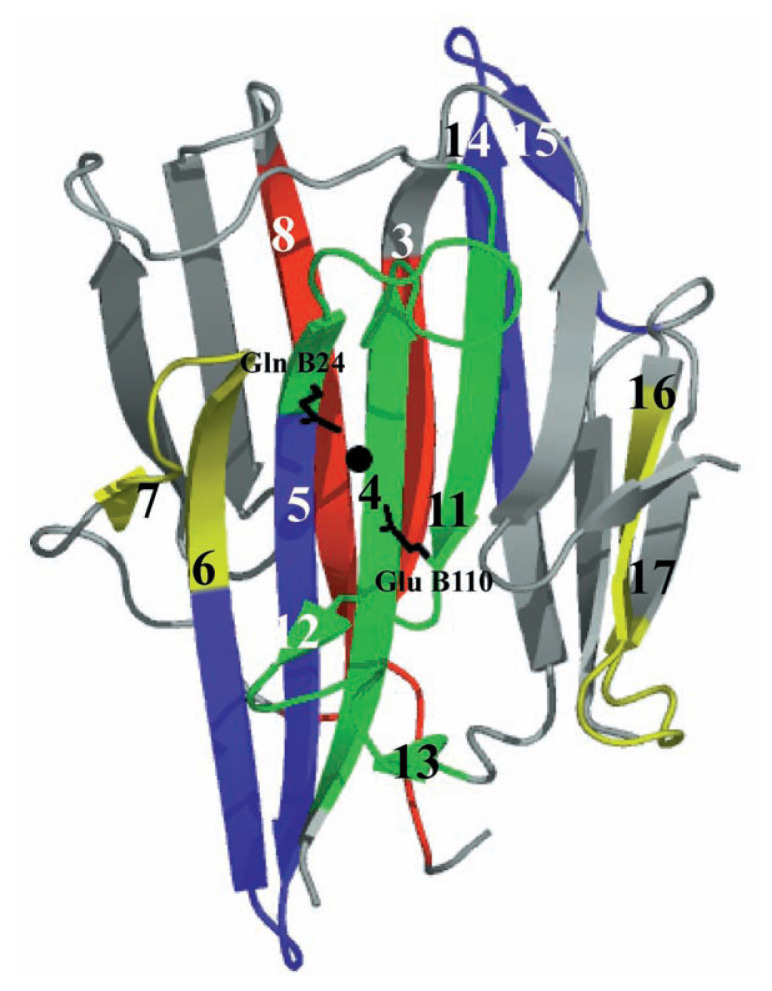
Apparent two-fold symmetry in the AGP molecule. The side chains of Glu B110 and Gln B24 and a water molecule sitting in between (a black ball) are also shown. A two-fold symmetry axis positioned between the antiparallel strands 4 and 11 passes near the residue Glu B110. Each typical symmetric pair of ***β***-strands is shown in the same color. The equivalent pairs are as follows: strand 3 corresponds to strand 8 (in red), strand 4 and the N-terminal part of strand 5 to strands 11, 12 and 13 (in green), the C-terminal part of strand 5 and the N-terminal part of strand 6 to the C-terminal part of strand 14 and strand 15 (in blue), the C-terminal part of strand 6 and strand 7 to strand 16 and the N-terminal part of strand 17 (in yellow). The gray parts do not have any relationships.

**Fig. 3:**
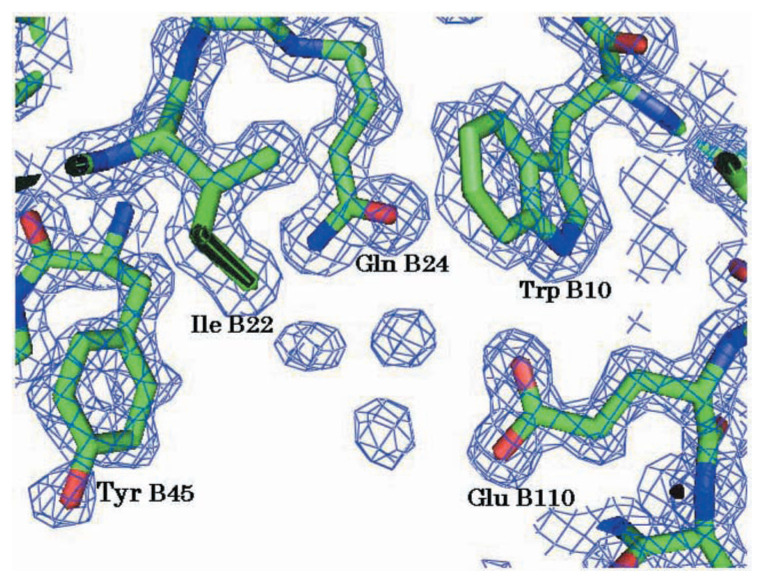
Electron density map and the corresponding chemical structure of the putative active site region of AGP. 2*F*_o_-*F*_c_ map is contoured at 1sigma.

Interestingly, there appears to be a weak, but significant partial two-fold symmetry in the structure of AGP, as shown in Fig. 2. There is a water molecule sitting in the center of the protein molecule, toward which the putative catalytic residues, Glu B110 and Gln B24, extend their side chains from the symmetric ***β***-strands 11 and 5, respectively (see also Fig. 3). These results might indicate the possibility that the AGP molecule was evolved by gene duplication and fusion as suggested more clearly for aspartic peptidases.17)

Figure 3 shows the electron density map and deduced structure of part of AGP including the putative catalytic residues, Glu B110 and Gln B24. These residues are separated by a closest distance of 4.8 Å between atoms Glu B110 O***ɛ***^1^ and Gln B24 O***ɛ***^1^, forming a hydrogen bond net work with the three nearby water molecules and Trp B10 is hydrogen-bonded to Glu B110. These results are essentially the same as those obtained with SGP,7) and therefore the mechanism of catalysis should be the same for both enzymes. The catalytic dyad composed of a glutamic acid and a glutamine is novel, and Fujinaga *et al*.7) have proposed a mechanism for SGP in which the glutamic acid residue acts as a general base to accept a proton from one of the water molecule, which in turn attacks nucleophilically the carbonyl carbon of the scissile peptide bond of the substrate, while the glutamine residue stabilizes the transition state through hydrogen bonding. Further studies will be necessary, however, to strictly establish their catalytic mechanism.
